# Structural and functional characterization explains loss of dNTPase activity of the cancer-specific R366C/H mutant SAMHD1 proteins

**DOI:** 10.1016/j.jbc.2021.101170

**Published:** 2021-09-04

**Authors:** Nicole E. Bowen, Joshua Temple, Caitlin Shepard, Adrian Oo, Fidel Arizaga, Priya Kapoor-Vazirani, Mirjana Persaud, Corey H. Yu, Dong-Hyun Kim, Raymond F. Schinazi, Dmitri N. Ivanov, Felipe Diaz-Griffero, David S. Yu, Yong Xiong, Baek Kim

**Affiliations:** 1 Department of Pediatrics, School of Medicine, Emory University, Atlanta, Georgia, USA; 2 Department of Molecular Biophysics and Biochemistry, School of Medicine, Yale University, New Haven, Connecticut, USA; 3 Department of Radiation Oncology, School of Medicine, Emory University, Atlanta, Georgia, USA; 4 Department of Microbiology and Immunology, Albert Einstein College of Medicine, Bronx, New York, USA; 5 Department of Biochemistry and Structural Biology, University of Texas Health San Antonio, San Antonio, Texas, USA; 6 School of Pharmacy, Kyung-Hee University, Seoul, South Korea; 7 Children's Healthcare of Atlanta, Atlanta, Georgia, USA

**Keywords:** SAMHD1, cancer, dNTPs, enzymology, structure, AGS, Aicardi–Goutières syndrome, dNTPase, dNTP triphosphohydrolase, FBS, fetal bovine serum, HA, hemagglutinin, HD, histidine–aspartate, IL-2, interleukin-2, IRF7, interferon regulatory transcription factor 7, ISRE, interferon-stimulated response element, LTR, long terminal repeat, NIH, National Institutes of Health, PDB, Protein Data Bank, PEI, polyethyleneimine, PHA, phytohemagglutinin, RIPA, radioimmunoprecipitation, RFP, red fluorescent protein, SAMHD1, sterile alpha motif and histidine–aspartate domain–containing protein 1, SIV, simian immunodeficiency virus, TP, triphosphate, VLP, virus-like particle, Vpx, viral protein X

## Abstract

Elevated intracellular levels of dNTPs have been shown to be a biochemical marker of cancer cells. Recently, a series of mutations in the multifunctional dNTP triphosphohydrolase (dNTPase), sterile alpha motif and histidine–aspartate domain–containing protein 1 (SAMHD1), have been reported in various cancers. Here, we investigated the structure and functions of SAMHD1 R366C/H mutants, found in colon cancer and leukemia. Unlike many other cancer-specific mutations, the SAMHD1 R366 mutations do not alter cellular protein levels of the enzyme. However, R366C/H mutant proteins exhibit a loss of dNTPase activity, and their X-ray structures demonstrate the absence of dGTP substrate in their active site, likely because of a loss of interaction with the γ-phosphate of the substrate. The R366C/H mutants failed to reduce intracellular dNTP levels and restrict HIV-1 replication, functions of SAMHD1 that are dependent on the ability of the enzyme to hydrolyze dNTPs. However, these mutants retain dNTPase-independent functions, including mediating dsDNA break repair, interacting with CtIP and cyclin A2, and suppressing innate immune responses. Finally, SAMHD1 degradation in human primary-activated/dividing CD4+ T cells further elevates cellular dNTP levels. This study suggests that the loss of SAMHD1 dNTPase activity induced by R366 mutations can mechanistically contribute to the elevated dNTP levels commonly found in cancer cells.

Sterile alpha motif (SAM) and histidine–aspartate (HD) domain–containing protein 1 (SAMHD1) is a dNTP triphosphohydrolase (dNTPase) that hydrolyzes dNTP substrates into their deoxynucleoside and triphosphate (TP) subparts (1, 2, 3). Mutations in SAMHD1 were first reported in patients with a rare genetic neuroimmunological disorder, Aicardi–Goutières syndrome (AGS) (4). Patients with AGS develop hyperactivation of the innate immune response in the absence of infection, which interferes with brain development and causes death at early ages (5). As proposed for other AGS-related proteins, such as three prime repair exonuclease 1 (6), RNase H2 (7), and adenosine deaminase 1 acting on RNA (8), SAMHD1 mutations may interrupt cellular nucleic acid metabolism, which can trigger hyperinterferon responses (9). In addition, SAMHD1 dNTPase activity restricts HIV-1 infection in nondividing myeloid cells (10, 11). SAMHD1-mediated intracellular dNTP depletion kinetically suppresses the reverse transcription step of HIV-1, which consumes intracellular dNTPs during proviral DNA synthesis (12, 13). SAMHD1 also inhibits the replication of other viruses (14, 15, 16, 17, 18) as well as retrotransposons (19, 20).

SAMHD1 harbors additional biological activities that are independent of the enzyme's dNTPase capability. First, SAMHD1 is involved in dsDNA break repair, which requires interaction with CtlP in order to promote DNA end resection and homologous recombination during repair (21). Next, SAMHD1 promotes cellular DNA replication by facilitating the removal of nascent DNAs at stalled replication forks by enhancing Mre11 nuclease (22). This process appears to reduce the synthesis of cellular nucleic acid products that can induce innate immunity activation (22). In addition, SAMHD1 suppresses the innate immune response by inhibiting NF-κB and interferon regulatory transcription factor 7 (IRF7) activation by reducing the phosphorylation of the NF-κB inhibitory protein, IκBα, and reducing inhibitor-κB kinase ε–mediated IRF7 phosphorylation, respectively (23, 24). SAMHD1 also blocks long terminal repeat (LTR)–mediated HIV-1 transcription (25), a process known to require NF-κB (26). Finally, SAMHD1 binds single-stranded nucleic acids, primarily when in the monomeric form (27, 28).

SAMHD1 activity is regulated at multiple levels. First, the enzymatically active SAMHD1 tetramer is formed when dGTP/GTP and dNTPs bind to two allosteric sites of SAMHD1, A1 and A2, respectively (29, 30, 31). Second, the phosphorylation of SAMHD1 residue T592 at the enzyme's C-terminal tail by cyclin A2/cyclic-dependent kinase 1/cyclic-dependent kinase 2 regulates HIV-1 restriction activity (32). However, phosphorylated inactive SAMHD1 becomes dephosphorylated by cellular PP2A–B55a phosphatase during mitotic exit of dividing cells (33). Finally, SAMHD1 restricts HIV-1 replication in nondividing myeloid cells such as macrophages and microglia, whereas HIV-2 and some simian immunodeficiency virus (SIV) strains effectively escape from SAMHD1 restriction by employing their accessory proteins, viral protein X (Vpx) or Vpr, to proteasomally degrade SAMHD1 and elevate dNTP levels (10, 11, 34), which enables these lentiviruses to replicate rapidly even in nondividing myeloid cells (13, 35).

Cancer cells harbor 6 to 11-fold higher intracellular dNTP levels than normal cells, and this dNTP elevation is a biochemical marker of cancer cells (36). Elevated intracellular dNTP level is mechanistically tied with uncontrolled/rapid cell division and higher cell population at S phase of cancer cells where dNTP biosynthesis is activated (37). Recently, a series of SAMHD1 mutations have been reported in various cancer cells including leukemias (38, 39, 40, 41, 42), lymphomas (43, 44), lung cancer (45), and colon cancer (46, 47, 48). However, it is unclear how these SAMHD1 mutations mechanistically contribute to cancer cell phenotypes. Importantly, as observed for SAMHD1 mutations in AGS cells (5, 49), SAMHD1 cancer-associated mutations are found throughout the entire protein, and most cause reduced SAMHD1 protein levels (39). These low protein levels could be indicative of structural alterations, which makes it difficult to investigate the mechanistic and functional alternations made by the SAMHD1 cancer mutations. In this report, we structurally and functionally investigated R366C/H SAMHD1 mutations, found in leukemia (42) and colon cancer (46, 47), but not in AGS. Notably, the R366C/H mutants possess protein stability profiles comparable to WT and retained all functions tested except dNTPase activity. This study suggests that SAMHD1 mutations can contribute to the intracellular dNTP level elevation commonly observed in cancer cells.

## Results

### R366C/H mutants are cancer-associated mutants with WT protein expression level

**Figure 1:**
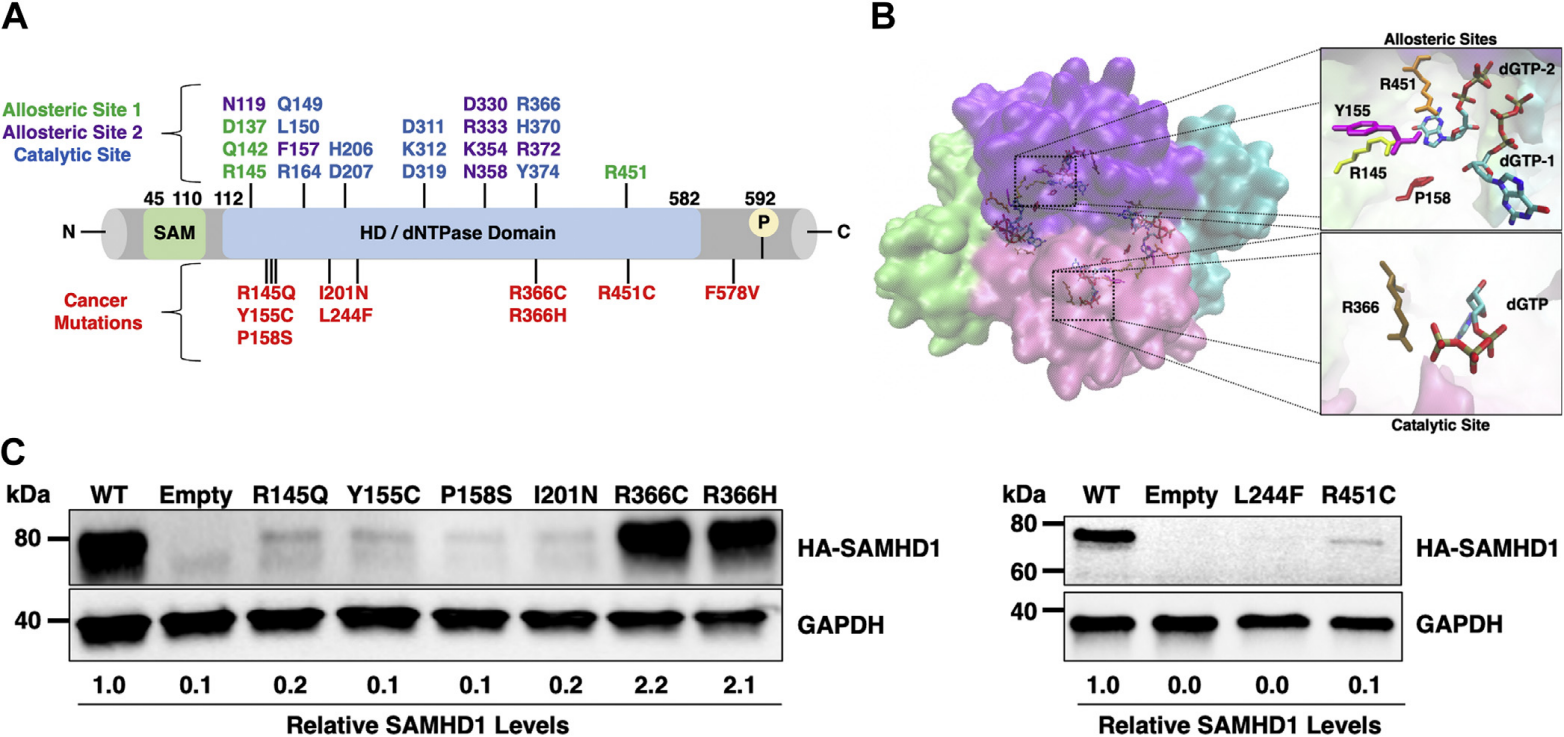
**Structural locations of selected SAMHD1 cancer mutations and their impact on intracellular SAMHD1 protein levels.***A*, linear map of SAMHD1 mutations selected in this study. Cancer-specific mutations are in *red* at the bottom, and residues in catalytic sites and two allosteric sites are marked in different colors. *B*, structural locations of SAMHD1 cancer mutations found in the allosteric or catalytic sites. Our previously solved WT HD domain tetramer structure (4BZB), which is bound to dGTP at its two allosteric sites (dGTP-1 and dGTP-2) and catalytic site, was used for locating the selected mutations. Subunits of the tetramers are displayed in different colors. *C*, the SAMHD1 protein levels in 293T cells transfected with an equal amount of plasmids expressing HA-tagged SAMHD1 proteins. Empty: backbone plasmids, pKH3-3xHA (*left*) and pLVX-IRES-mCherry (*right*). Transfection efficiency was determined by GFP expression from the cotransfected eGFP control plasmid (*left*) or from mCherry expression (*right*) by flow cytometry (Table S1). The relative SAMHD1 protein levels were normalized by GAPDH protein level, and the ratios between WT and mutant SAMHD1 protein levels were calculated. HA, hemagglutinin; HD, histidine–aspartate; SAMHD1, sterile alpha motif domain and histidine–aspartate domain–containing protein 1.

To investigate the impact of cancer-associated mutations on SAMHD1 functions, we searched for SAMHD1 mutants showing WT-level cellular protein stability. For this, we conducted a screen of multiple SAMHD1 mutants for their cellular stability in 293T cells transfected with an equal amount of SAMHD1 expression plasmids (Fig. 1*A*). SAMHD1 mutants selected for the screen include leukemia-associated mutants (39), R145Q, Y155C, P158S, and R451C, which are located in the allosteric sites of the enzyme (Fig. 1*B*). R366C, found in both colon cancer (47) and leukemia (42), and R366H, found in colon cancer (46), are located in the enzyme's catalytic site (Fig. 1*B*). Notably, the R366 residue coordinates the γ-phosphate group of the substrate dNTP in the catalytic pocket. Finally, I201N and L244F, found in leukemia (39), are located outside both these functional domains (Fig. 1*A*). As shown in an immunoblot assay with the transfected 293T cells (Fig. 1*C*), R145Q, Y155C, P158S, I201N, L244F, and R451C displayed a marked reduction in expression level when compared with WT SAMHD1, suggesting their cellular protein instability. This finding is consistent with previous reports, which show decreased SAMHD1 expression in primary cells harboring SAMHD1 mutations isolated from leukemia patients (39). Importantly, the R366C/H mutants show similar expression levels to WT SAMHD1. Mutants from this initial screen that have poor cellular expression profiles likely possess several functional impairments because of their general protein instability. However, the unaltered cellular expression of R366C and R366H allowed us to use these mutants as a tool for investigating which functions of SAMHD1 may be implicated in the enzyme's role in cancer cells.

### Biochemical analyses of protein stability and structural integrity of R366C/H mutants

**Figure 2:**
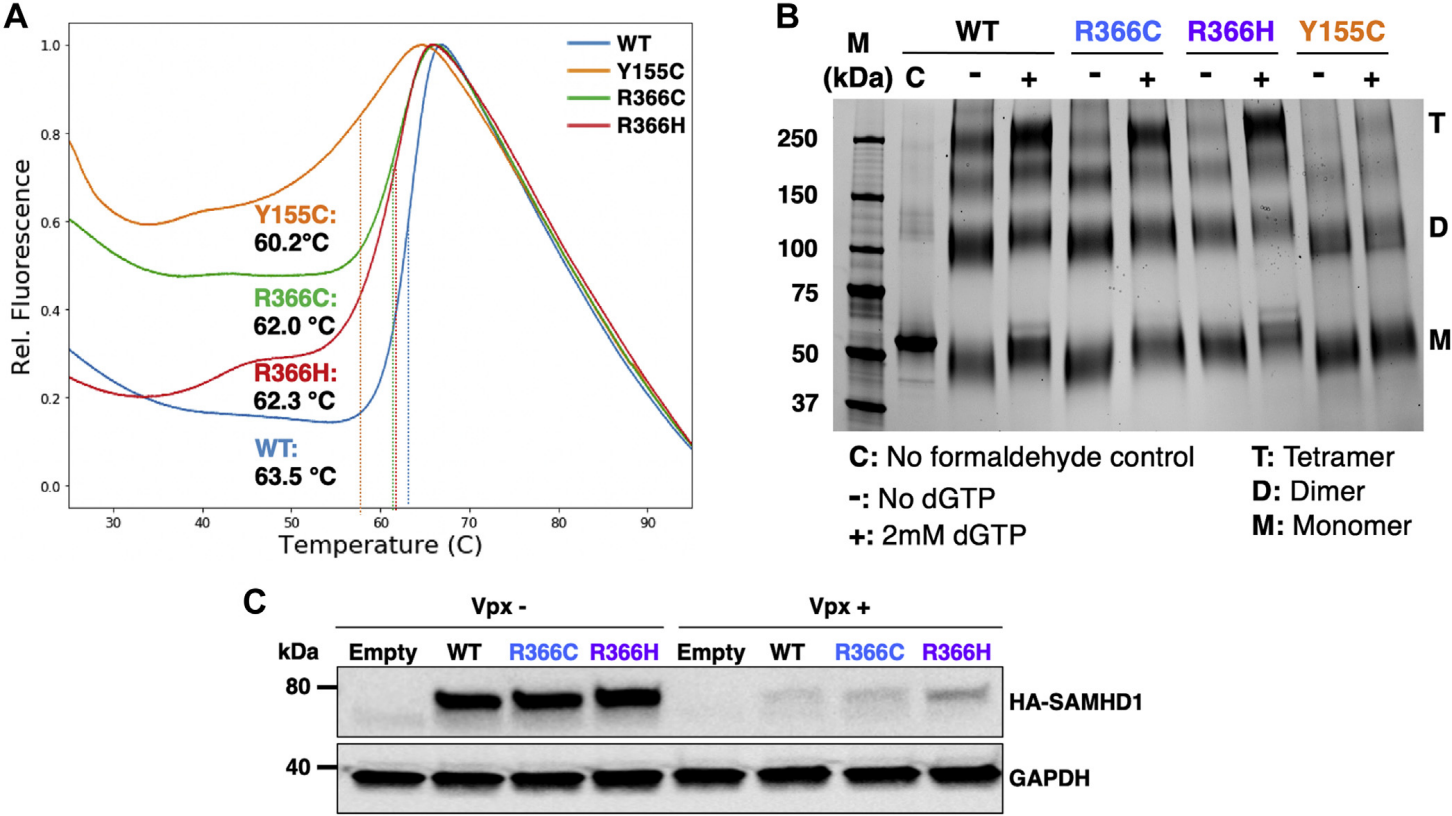
**Thermostability, tetramerization, and Vpx-mediated degradation of SAMHD1 mutant proteins.***A*, thermal shift assay of WT and mutant HD proteins was conducted after preincubation with SYPRO Orange dye. The *T*_m_ of each protein was calculated as described in the Experimental procedures section: *T*_m_: WT = 63.5 ± 0.1 °C, Y155C = 60.2 ± 0.3 °C, R366C = 62.0 ± 0.4 °C, R366H = 62.3 ± 0.1 °C. *B*, tetramerization of WT and mutant HD domain proteins was analyzed by SDS-PAGE after formaldehyde-mediated crosslinking in the presence and absence of 2 mM dGTP. M: monomer, D: dimer, T: tetramer. M: molecular weight markers. C: no formaldehyde control. *C*, Vpx-mediated proteasomal degradation of WT and mutant SAMHD1 proteins in cells was monitored. 293T cells were cotransfected with SAMHD1-expressing plasmids and Vpx-expressing (Vpx+) or nonexpressing (Vpx−) plasmid. SAMHD1 protein levels in the transfected cells were determined by immunoblot with anti-SAMHD1 antibody. Vpx, viral protein X.

In order to further probe the overall protein stability changes induced by the R366C/H mutations, we overexpressed WT and mutant SAMHD1 proteins in *Escherichia coli* and purified these proteins for several biochemical and biophysical analyses. First, we employed a thermal shift assay to monitor the temperature-dependent stability of the SAMHD1 proteins. For this, each protein was incubated with activator dGTP and SYPRO Orange, and denaturation was monitored by increased relative fluorescence, as a function of temperature (Fig. 2*A*). Y155C, which displayed reduced cellular expression levels in 293T cells (Fig. 1*C*), exhibited an altered thermal shift curve and *T*_m_. Conversely, R366C and R366H displayed only minor alterations in thermal shift curves and melting temperatures. These minor alterations are not sufficient to induce changes in cellular protein level (Fig. 1*C*). As mutants that showed altered cellular protein stability had alterations in the allosteric site residues, it is possible that these stability changes are due to an inability to form the more stable tetramer conformation. To understand how differences in ability to form tetramers might underpin the stability changes provoked by these mutations, we utilized a cross-linking assay (50). Purified proteins were incubated with activator dGTP in order to induce tetramerization, and formaldehyde cross-linked products were analyzed using SDS-PAGE (Fig. 2*B*). The two stable R366C/H mutants were able to form tetramers comparable to WT SAMHD1. However, Y155C, representative of an unstable mutant, was unable to form tetramers. This suggests that the inability to form tetramers in the presence of dGTP activator may lead to the instability displayed by some cancer mutants. However, these data indicate that the R366C/H mutations do not impair oligomerization, further supporting the intact structural integrity of the two R366C/H mutant proteins.

HIV-2 and SIV code for Vpx, which targets SAMHD1 for proteolytic degradation, thus relieving restriction in nondividing cells (10, 11, 51). As the Vpx-binding site on SAMHD1 is dependent on the enzyme's three-dimensional structure (52), we utilized a Vpx degradation assay to further probe changes in structure (15). For this test, we transiently cooverexpressed Vpx with WT or R366C/H SAMHD1 in 293T cells and monitored SAMHD1 degradation using immunoblot (Fig. 2*C*). WT SAMHD1 was successfully degraded by Vpx, and R366C/H was comparably degraded. Overall, the multiple biochemical and cellular analyses validate that the three-dimensional structure of these R366C/H mutants is relatively preserved.

### Cancer-associated SAMHD1 mutants have significantly reduced dNTPase activity

**Figure 3:**
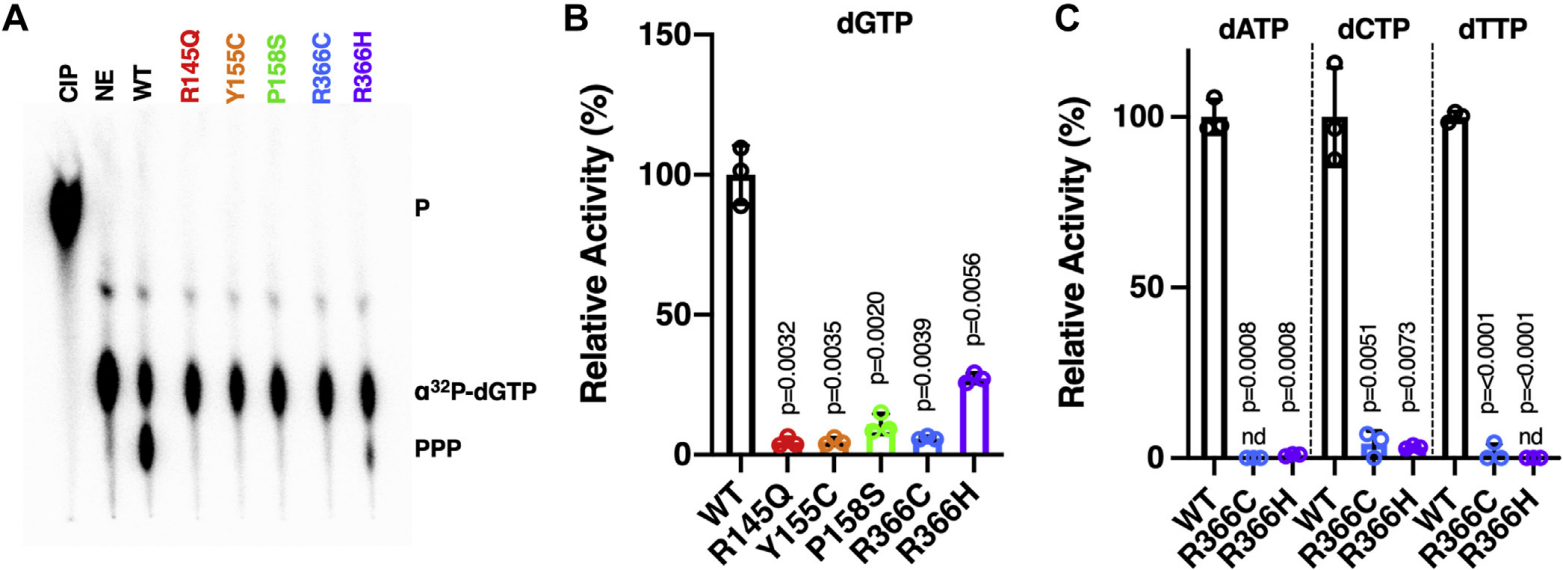
**Biochemical dNTPase activity comparison of WT and cancer mutant SAMHD1 proteins.***A*, TLC-based dNTPase assay was conducted using the same concentration of purified HD domain protein for each mutant. This assay monitors the production of triphosphate from α^32^P-dNTP substrate under the condition that triphosphate product generation was linear to protein amounts at the saturating dNTP substrate concentration. The reactions were analyzed by TLC as described in the Experimental procedures section. *B*, relative dGTPase activity of WT and mutant SAMHD1 proteins (HD domain) was calculated by dividing the triphosphate product by the lane total and normalizing to WT dGTPase activity. *C*, the relative dNTPase activities of WT, R336C, and R366H proteins were determined for dATP, dCTP, and dTTP. Data are the mean of three replicates, and error bars reflect standard deviation from the mean. *p* values were determined using two-tailed and unpaired Welch's *t* test with WT. CIP, calf-intestinal phosphatase control; nd, not detected; NE, no enzyme negative control; P, monophosphate; PPP, triphosphate.

SAMHD1 is a dNTP triphosphohydrolase that degrades dNTPs into their deoxynucleoside and TP components (1, 2, 3). As dNTP levels in cancer cells are 6 to 11-fold higher than that of normal cells (36), we hypothesized that cancer-associated SAMHD1 mutants would have reduced dNTPase activity, ultimately elevating intracellular dNTP levels. To test the dNTPase activity of SAMHD1 cancer mutants, we purified the catalytic core HD domain (residues 113–626) of some SAMHD1 mutants including R366C/H. We then incubated the purified enzymes with [α-^32^P]dGTP in the presence of excess unlabeled dGTP (31). dGTP not only binds to the two allosteric sites of SAMHD1 for activation but also serves as a substrate (1, 2). Production of labeled TP product (PPP) was monitored by a TLC-based dNTPase assay (Fig. 3*A*). A no-enzyme control and a calf intestinal phosphatase control were used to detect [α-^32^P]dGTP substrate and monophosphates (*P*), respectively. Incubation times were chosen such that product formation was in the linear range under saturating dNTP concentrations. Percent dGTP hydrolysis was calculated by dividing the TP product by the lane total (Fig. S1). Relative dGTPase activity was calculated by subtracting the no-enzyme control condition from percent hydrolysis and normalizing to WT. Interestingly, the dGTPase activity of R145Q, Y155C, P158S, and R366C is severely impaired compared to WT SAMHD1 activity (Fig. 3*B*). R366H possesses significantly lower dGTPase activity than WT but is more active than R366C. It is possible that the substitution of a positively charged histidine residue at position 366 is able to mediate minor coordination of the negatively charged γ-phosphate group of the substrate dNTP, whereas cysteine cannot mediate this interaction.

Given the R366C/H mutants displayed unaltered cellular protein levels but possessed reduced dGTPase activity, we performed a complete analysis of the dNTPase activity of these mutants. Therefore, we conducted the TLC-based dNTPase assay with the three remaining radioactive dNTP substrates (dATP, dCTP, and dTTP), where the unlabeled substrate and activator dGTP were in excess (Fig. 3*C*). Both the R366C and R366H mutants showed reduced dNTPase activity when incubated with each dNTP tested in comparison to WT SAMHD1. Together with the unaltered cellular protein level of R366C and R366H, this significantly impaired hydrolysis activity suggests that alterations in the dNTPase activity of SAMHD1 may mechanistically involve the enzyme in the elevated dNTP pools observed in cancer cells.

### X-ray crystal structures of R366C/H mutants

**Figure 4:**
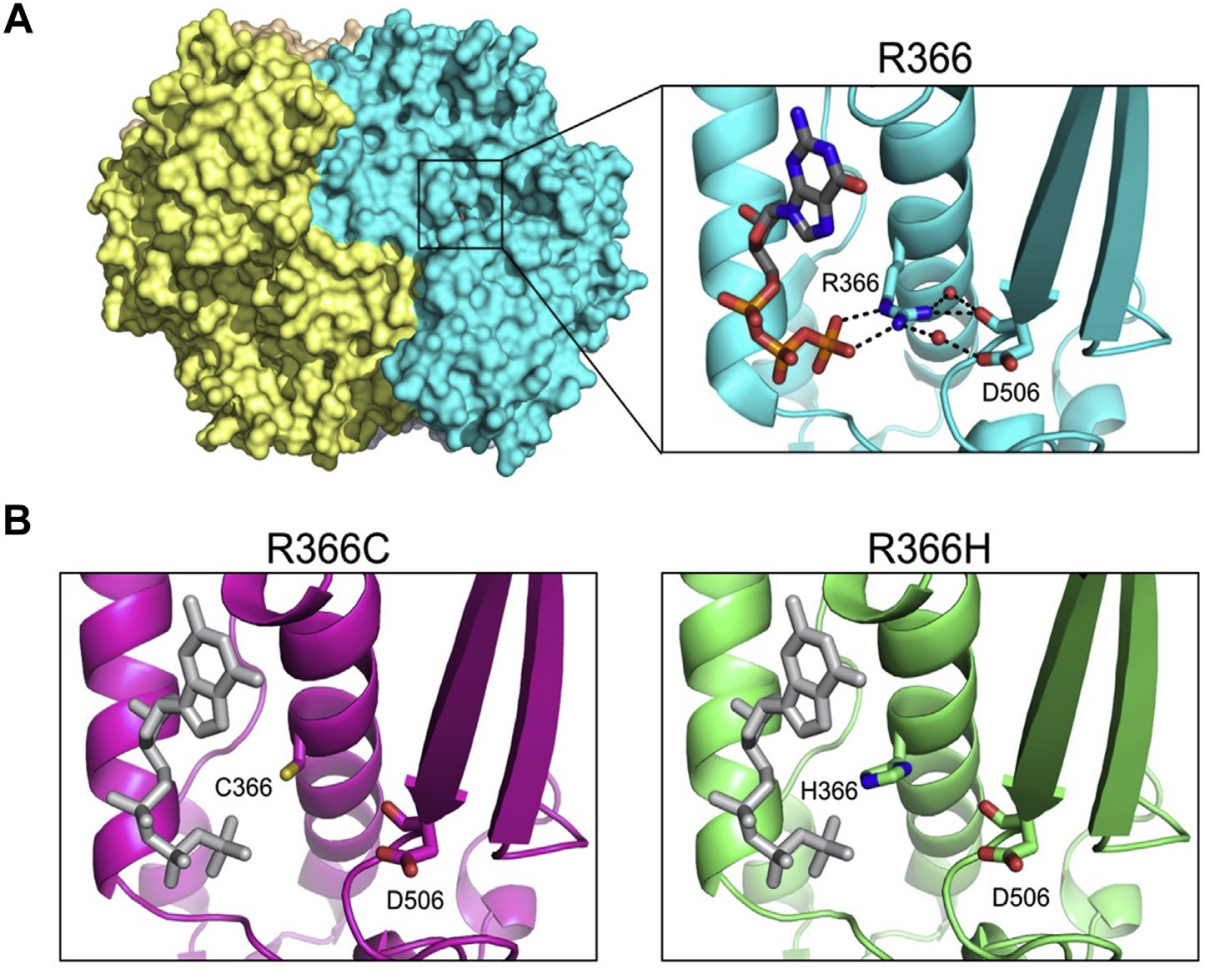
**R366C/H mutations abrogate catalytic nucleotide binding.***A*, overall structure of the SAMHD1 tetramer in surface representation (*left*). The *right inset* shows selected interactions of R336 and the catalytic nucleotide in SAMHD1_HD/RN_. H-bonds and salt bridges are shown as *dashed lines*. *B*, R336C (*left*) or R366H (*right*) leads to the disruption of the interactions and the loss of nucleotide binding at the catalytic site. The catalytic nucleotides (*gray*) are modeled based on their positions in SAMHD1_HD/RN_. Portions of the structure have been hidden for clarity. SAMHD1, sterile alpha motif domain and histidine–aspartate acid domain–containing protein 1.

For both the R366C and R366H mutants, we crystallized the HD domain of SAMHD1 (residues 113–626) in the presence of dGTP, which is capable of binding both allosteric sites 1 and 2 as well as the catalytic site of SAMHD1 (29). In the catalytic site of WT protein, the R366 guanidinium group neutralizes the negative charge of the substrate dNTP γ-phosphate and interacts with D506 to stabilize the bound nucleotide (Fig. 4*A*). An established catalytically inactive mutant (H206R/D207N; SAMHD1_HD/RN_) was used to prevent hydrolysis but retain binding capacity of dGTP during crystallization (29). Crystals for R366C and R366H diffracted to a resolution of 1.9 and 3.6 Å, respectively (Table S2).

Inspection of each R366 mutant structure shows neither mutation impairs oligomerization or induces significant deviations from its tetramer structure as compared with a previously solved dGTP-bound SAMHD1_HD/RN_ tetramer (RMSD_R366C_: 0.36 Å and RMSD_R366H_: 0.39 Å when compared with Protein Data Bank [PDB]: 4BZB). However, unlike WT protein structure that displays dGTP substrate bound to the catalytic site, no nucleotide density was observed in the catalytic pocket of either mutant (Figs. 4*B* and S2), thus confirming that substitution of a cysteine or histidine side chain at residue 366 renders SAMHD1_HD/RN_ deficient in stably binding dGTP at the catalytic site despite tetramerization. Uniquely, when dGTP was modeled to fit the catalytic site of R366H mutant, N ε2 of the mutant R366H side chain sits close to the γ-phosphate position in a modeled dGTP-bound structure and is therefore theoretically capable of an interaction, which could explain the higher relative R366H enzymatic activity for dGTP *versus* other mutants (Fig. 2*B*).

### Impact of the R366C/H mutation on SAMHD1 restriction of HIV-1

**Figure 5:**
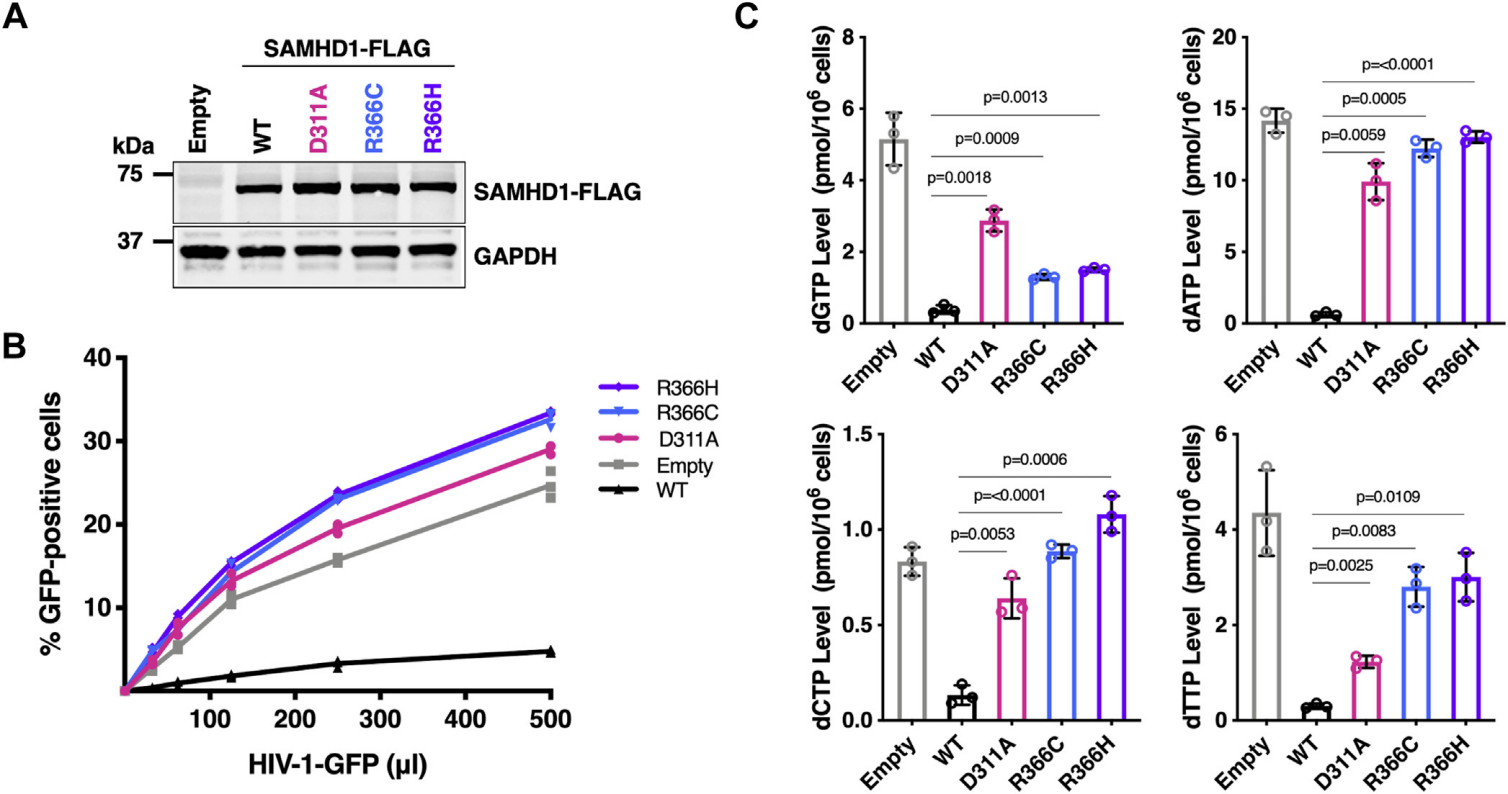
**HIV-1 restriction capability of R366H/C SAMHD1 mutants**. *A*, U937 cells were transduced with lentiviral vector expressing FLAG-tagged WT, D311A inactive mutant, R366C, and R366H SAMHD1 proteins. The expression level of each SAMHD1 protein was determined by Western blot with anti-FLAG antibody and normalized with GAPDH. *B*, the transduced U937 cells were differentiated to nondividing macrophage stage and transduced with eGFP-expressing HIV-1 vector. Transduction efficiency using different quantities of HIV-1 vector was determined using flow cytometry. *C*, intracellular dNTP levels in differentiated U937 cells expressing WT and mutant SAMHD1 proteins were determined by RT-based dNTP assay. Data are the mean of three replicates, and error bars reflect standard deviation from the mean. *p* values were determined using two-tailed and unpaired Welch's *t* test to WT knock-in cells. SAMHD1, sterile alpha motif domain and histidine–aspartate acid domain–containing protein 1.

SAMHD1 restricts HIV-1 replication in nondividing cells by impairing reverse transcription (12, 13). As this restriction activity correlates to the dNTPase activity of SAMHD1, we predicted that the R366C/H mutants would be unable to restrict HIV-1. To examine this, we utilized U937 cells, which lack endogenous SAMHD1 (11). U937 cells were transduced to express WT or R366C/H SAMHD1, and their equal cellular protein levels were confirmed with an immunoblot (Fig. 5*A*). SAMHD1 D311A active site mutant was used as a negative control as this mutant is known to have dNTPase and HIV-1 restriction deficits (53). We differentiated the transduced U937 cells into nondividing macrophages and challenged with increasing amounts of HIV-1-GFP vector (Fig. 5*B*) (49). While expression of WT SAMHD1 was sufficient to restrict HIV-1-GFP transduction, the R366C/H mutants were unable to restrict transduction, similar to the D311A mutant. Next, we measured the dNTP levels of these differentiated cell lines using a reverse transcription-based single-nucleotide incorporation assay (Fig. 5*C*) (54). Consistent with our biochemical dNTPase activity characterization, cells expressing R366C and R366H had raised intracellular dNTP levels when compared with cells expressing WT SAMHD1, which is responsible for the failed restriction against HIV-1. Importantly, this elevation of dNTP pools in cells expressing R366C/H SAMHD1 mimics the cancer cell phenotype.

### R366C/H mutants have unaltered interactions with CtIP for dsDNA break repair and cyclin A2

**Figure 6:**
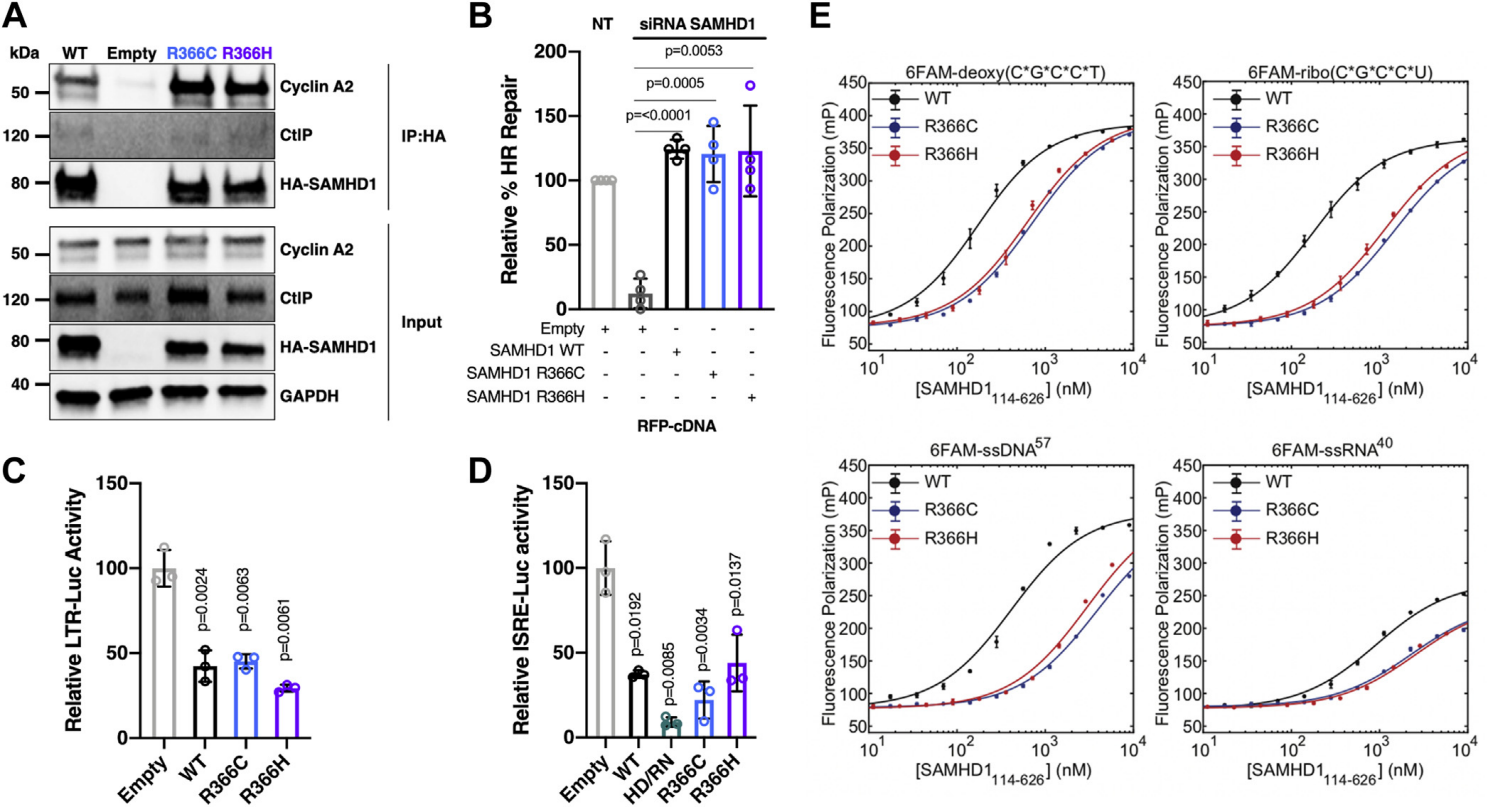
**Effect of R366C and R366H SAMHD1 mutations on SAMHD1 dNTPase-independent functions.***A*, immunoprecipitation of cyclin A2 and CtlP nuclease by SAMHD1. 293T cells were transfected with an equal amount of HA-tagged SAMHD1-expressing plasmids, and the expressed SAMHD1 proteins were pulled down by HA antibody beads. The lysates (input) of the transfected cells and the pull-down fractions (IP:HA) were assessed by immunoblot using anti-HA antibody, cyclin A2 antibody, and CtlP antibody. GAPDH was used for the loading control. Empty: empty plasmid (pLVX-IRES-mCherry). *B*, dsDNA break repair activity of WT and R366C/H mutants. U20S cells with integrated DR-GFP reporter construct were transfected with I-Sce-I, SAMHD1-UTR siRNA, and RFP-SAMHD1 WT, or R366C/H cDNA. Flow cytometry was used to assess for homologous recombination, as measured by GFP expression in cells expressing RFP. Empty: RFP plasmid. *C*, suppression of LTR-driven gene expression by WT and R366C/H mutants. 293T cells were transfected with SAMHD1-expressing plasmids, HIV-1 LTR-firefly luciferase plasmid (LTR-Luc), and Renilla luciferase plasmid as a transfection control. Firefly luciferase activity was measured and normalized to Renilla luciferase activity 24 h post-transfection. *D*, suppression of ISRE stimulation by WT and R366C/H mutants. 293T cells were transfected by SAMHD1-expressing plasmids, ISRE-firefly luciferase plasmid (ISRE-Luc), IRF7 plasmid, and Renilla luciferase plasmid as a transfection control. Firefly luciferase activity was measured and normalized to Renilla luciferase activity 24 h post-transfection. *E*, nucleic acid–binding curves and binding constants of WT and two R366 mutant SAMHD1 proteins were determined using fluorescence polarization. 6FAM-deoxy(C∗G∗C∗C∗T): WT *K*_*d*_ = 146.8 ± 8.3 nM, R366C *K*_*d*_ = 689 ± 39 nM, R366H *K*_*d*_ = 606 ± 46 nM. 6FAM-ribo(C∗G∗C∗C∗U): WT *K*_*d*_ = 153.8 ± 4.2 nM, R366C *K*_*d*_ = 1449 ± 51 nM, R366H *K*_*d*_ = 1125 ± 42 nM. 6FAM-ssDNA^57^: WT *K*_*d*_ = 387.2 ± 45.4 nM, R366C *K*_*d*_ = 3825 ± 340 nM, R366H *K*_*d*_ = 2965 ± 328 nM. 6FAM-ssRNA^40^: WT *K*_*d*_ = 884.8 ± 59.9 nM, R366C *K*_*d*_ = 2252 ± 249 nM, R366H *K*_*d*_ = 2476 ± 264 nM. Data are the mean of three replicates, and error bars reflect standard deviation from the mean. *p* values were determined using two-tailed and unpaired Welch's *t* test to *B*. SAMHD1 siRNA + empty, *C*, empty, *D*, empty. dNTPase, dNTP triphosphohydrolase; HA, hemagglutinin; ISRE, interferon-stimulated response element; LTR, long terminal repeat; RFP, red fluorescent protein; SAMHD1, sterile alpha motif domain and histidine–aspartate domain–containing protein 1.

SAMHD1 has multiple dNTPase-independent functions including interacting with cell cycle proteins (55), mediating dsDNA break repair (21), restricting transcription from the HIV-1 proviral LTR (25), suppression of the innate immune response (17, 24), and nucleic acid binding (27). Thus, we investigated whether these functions were preserved in the R366C/H mutants. First, we tested binding to cell cycle protein, cyclin A2, by overexpressing hemagglutinin (HA)-SAMHD1 or HA-SAMHD1 R366C/H in 293T cells and performing a coimmunoprecipitation (Fig. 6*A*). Immunoprecipitation of both R366C and R366H SAMHD1 pulled down cyclin A2 similarly to WT SAMHD1, indicating binding to cell cycle proteins is maintained by these mutants. SAMHD1 was recently implicated in dsDNA break repair by homologous recombination through its interaction with CtIP (21). To test binding of the R366C/H mutants to CtIP, we probed for CtIP in the HA-SAMHD1 pulldowns described previously (Fig. 6*A*). Similarly to cyclin A2, we found that R366C and R366H maintained binding to CtIP.

Next, we investigated the ability of the R366C/H mutants to mediate dsDNA damage repair by homologous recombination. For this, we utilized U20S cells containing an integrated direct-repeat GFP construct with an I-SceI cut site (21). Upon addition of I-SceI endonuclease, a dsDNA break is generated that, when repaired by homologous recombination, results in a functional GFP construct. We knocked down endogenous SAMHD1 using siRNA and expressed either siRNA-resistant red fluorescent protein (RFP)-WT or RFP-R366C/H SAMHD1 (Fig. S3*A*). Therefore, the ability of the mutants to promote homologous recombination can be determined by monitoring GFP expression in RFP-expressing cells. As shown in Figure 6*B*, both R366C and R366H were able to mediate homologous recombination similar to WT SAMHD1, indicating that R366C/H maintain the dNTPase-independent dsDNA damage repair function.

### R366C/H mutants suppress HIV-1 LTR activation and innate immune activation

In addition to restricting replication at the reverse-transcription step, SAMHD1 has been implicated in suppressing viral transcription from the HIV-1 LTR (25). We tested the ability of the dNTPase-impaired R366C/H mutants to suppress expression from the proviral LTR by cotransfecting 293T cells with WT or mutant SAMHD1 expression plasmids and an HIV-1 LTR-Luciferase construct (Fig. 6*C* and Fig. S3*B*). As observed with WT SAMHD1, the R366C and R366H mutants were able to suppress LTR-driven luciferase production.

SAMHD1 suppresses the innate immune response in dividing cells independent of dNTPase activity through interactions with IRF7 and NF-κB (17, 24). To assess for alterations in the ability of R366C/H to suppress the innate immune response, we transiently coexpressed firefly luciferase under the control of an interferon-stimulated response element (ISRE-Luc), transfection control Renilla luciferase, IRF7-FLAG, and SAMHD1 WT, HD/RN, R366C, or R366H in 293T cells (Fig. S3*C*). Twenty-four hours after transfection, firefly luciferase activity was assessed as a measure of innate immune response stimulation and normalized to Renilla luciferase activity (Fig. 6*D*). As anticipated, both WT and HD/RN SAMHD1 suppressed firefly luciferase activity in this assay. R366C and R366H also displayed suppressed firefly luciferase. Thus, the R366C/H mutations do not alter the SAMHD1 function for innate immune response suppression in dividing cells.

### R366C/H mutants showed reduced nucleic acid–binding activity

SAMHD1 has been shown to bind nucleic acids, which seem to play a role in HIV-1 restriction (27, 28). We investigated whether the R366C/H mutations induced changes in nucleic acid binding using a fluorescent polarization assay. R366C and R366H showed reduced nucleic acid–binding affinity in this assay (Fig. 6*E*). It is possible that this is a consequence of overlap between the dNTPase catalytic site and the nucleic acid–binding domain (28). However, it is unclear at present that this altered nucleic acid–binding activity has a connection to cancer cell phenotypes.

### SAMHD1 reduction in human primary normal dividing cells further elevates intracellular dNTP levels

**Figure 7:**
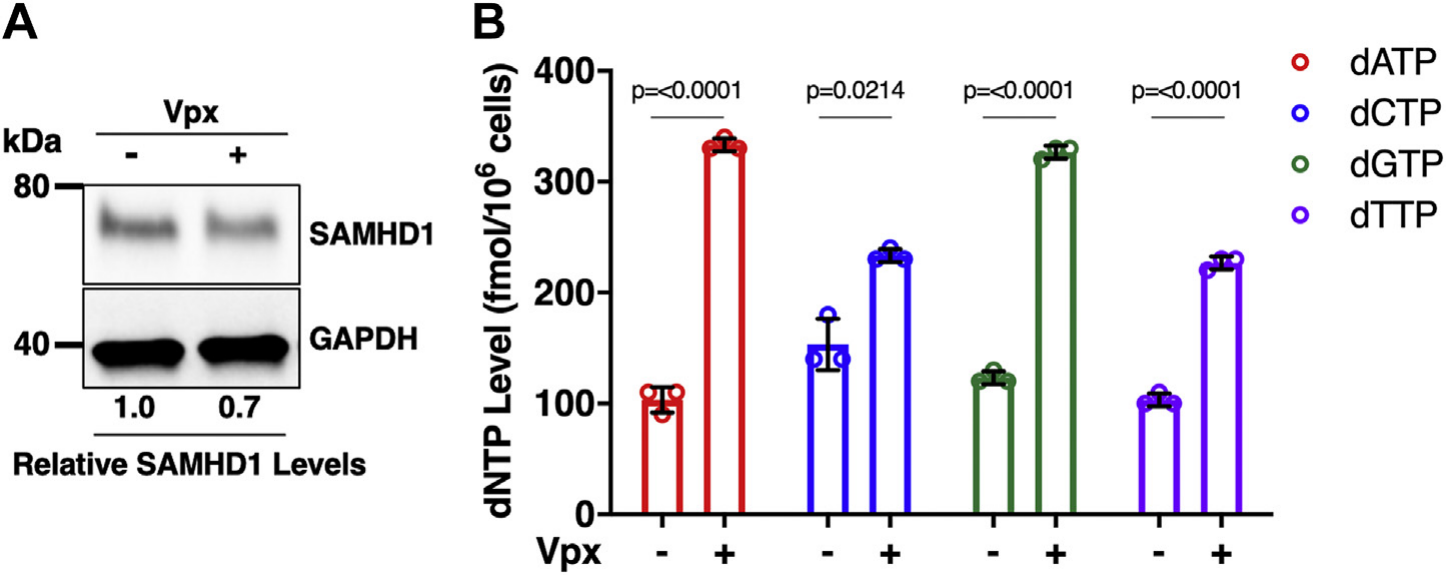
**Effect of SAMHD1 degradation on intracellular dNTP levels in human primary-activated/dividing CD4+ T cells.** Human primary CD4+ T cells were isolated from three healthy donors, activated by PHA and IL-2 for 5 days, and treated with VLP Vpx (−) or VLP Vpx (+) for 24 h *A*, cellular SAMHD1 protein levels were determined by immunoblot using anti-SAMHD1 antibody and anti-GAPDH antibody as a loading control. The relative SAMHD1 protein levels were normalized by GAPDH protein level, and the ratios between WT and mutant SAMHD1 protein levels were calculated. *B*, intracellular dNTP levels were determined by RT-based dNTP assay. Data are the mean of three replicates, and error bars reflect standard deviation from the mean. *p* values were determined using two-tailed and unpaired Welch's *t* test to VLP Vpx (-) condition. IL-2, interleukin-2; PHA, phytohemagglutinin; SAMHD1, sterile alpha motif domain and histidine–aspartate domain–containing protein 1; VLP, virus-like particle.

Finally, we sought to evaluate the contribution of SAMHD1 alterations in driving elevated dNTP levels, a key molecular signature of cancer, in a primary cell model. To this end, we obtained activated/dividing CD4^+^ T cells positively selected from peripheral blood mononuclear cells of three healthy donors and stimulated with phytohemagglutinin (PHA) and interleukin-2 (IL-2) media for 3 days. In fact, we previously reported that human primary activated CD4+ T cells already harbor high dNTP concentration (54). Furthermore, these primary dividing cells also express SAMHD1 as shown in Figure 7*A*. To test whether SAMHD1 level decrease can elevate dNTP concentration in activated CD4^+^ T cells, we treated these CD4+ T cells with virus-like particles (VLPs) containing Vpx (VLP Vpx+) to degrade endogenous SAMHD1 or VLP Vpx− as a negative control. We then measured intracellular dNTP levels of these treated cells. As shown in Figure 7*A*, even though the proportion of SAMHD1 degraded was small, 30%, we observed a significant increase in dNTP levels in the cells treated with VLP Vpx+, compared with the cells treated with VLP Vpx− (Fig. 7*B*). These data suggest that, in the context of a primary cell model, reduction in SAMHD1 protein level, which also reduces overall dNTP hydrolysis capacity, can induce the elevated dNTP levels commonly seen in cancer cells.

## Discussion

The sole utility of intracellular dNTPs is DNA synthesis, whereas rNTPs harbor highly versatile utilities in cells, including RNA synthesis, kinase substrates, and energy carriers. Intracellular dNTP concentrations (single-digit millimolar) are several hundred times lower than rNTP concentrations (close to millimolar range) (56), and dNTP biosynthesis is tightly regulated during cell cycle in dividing cells (57, 58). However, elevated dNTP levels are biochemical markers of cancer cells (36), likely because the uncontrolled cell division observed in these cells requires an abundant dNTP supply. dNTP biosynthesis pathways are extensively investigated, particularly in the field of cancer biology, which led to discovery of various anticancer therapeutics targeting dNTP biosynthesis pathways (59). However, the understanding of cellular dNTP degradation and hydrolysis mechanisms was relatively limited until the discovery of SAMHD1 dNTPase (1, 2). Recent identification of mutations in SAMHD1 in various cancer cell types generated a unique opportunity to understand the role of dNTP hydrolysis and its regulation in the elevated intracellular dNTP pools observed in cancer cells.

A majority of the SAMHD1 cancer-associated mutations tested were found to be structurally destructive, consistent with SAMHD1 mutations observed in patients with AGS (5, 49). However, R366C/H mutations, which were identified in two different cancer cell types (42, 46, 47), clearly do not affect the overall structural integrity essential for its various biological functions, enabling us to directly link the biochemical change induced by these mutations to a potential functional role of SAMHD1 in cancer cells. Indeed, among all biological and biochemical assays related to SAMHD1 activities and functions that were investigated, the loss of dNTPase activity is clearly the sole and prominent defect induced by R366C/H mutations. The loss of the interaction with γ-phosphate of the dNTP substrate at the catalytic site is directly supported by our observation that dGTP is absent in the catalytic site of the crystal packs of both R366C and R366H SAMHD1 tetramers even though we observed two dGTP molecules bound to the two allosteric sites in the same crystals.

Interestingly, several studies reported another role of SAMHD1 in anticancer therapeutics. We and others reported that SAMHD1 hydrolyzes ara-CTP (60, 61), and more interestingly, that cellular SAMHD1 protein levels are corelated with cellular araC resistance (62, 63). Clearly, SAMHD1, which hydrolyzes both araCTP and dCTP, a competitor of araCTP, can regulate the balance between the steady-state cellular levels of araCTP and dCTP that is important for araC anticancer efficacy.

It remains unclear whether the genetic loss of SAMHD1 in AGS is corelated with cancers, mainly because of the rare incidence and early death of patients with this severe immune disorder (5). Interestingly, SAMHD1 KO mice did not display an AGS phenotype (64, 65) and there is no report of cancer development in SAMHD1 KO mice, indicating that SAMHD1 functions may be species dependent. Overall, our study reveals that loss of dNTPase activity induced by SAMHD1 R366 cancer mutations can mechanistically contribute to the elevated intracellular dNTP pools commonly observed in cancer cells.

## Experimental procedures

### Cell culture

293T cells were cultured in Dulbecco's modified Eagle's medium (Gibco) supplemented with 10% (v/v) fetal bovine serum (FBS) and 1% (v/v) penicillin/streptomycin. U20S cells were grown in Dulbecco's modified Eagle's medium supplemented with 7.5% FBS. U937 cells were grown in RPMI (Corning) supplemented with 10% (v/v) FBS and 1% (v/v) penicillin/streptomycin. Primary human CD4+ T cells were isolated from monocyte-depleted peripheral blood buffy coats of three donors (New York Blood Center, New York City, New York) by positive selection using anti-CD4 antibody–conjugated magnetic beads as previously described (54). CD4+ T cells were activated by culturing in RPMI supplemented with 10% (v/v) FBS, 1% (v/v) penicillin/streptomycin, 5 μg/ml PHA (Sigma), and 5 ng/ml IL-2 (Miltenyi) for 5 days.

### Structural model with location of mutant residues

Visual Molecular Dynamics (University of Illinois at Urbana-Champaign), version 1.9.4, was used to visualize the structure of WT SAMHD1 bound to dGTP (PDB: 4BZB (29)) to identify the location of selected mutations.

### Mutant cellular expression

Plasmids expressing SAMHD1 cancer-associated mutations were generated by QuikChange (Agilent), using either pKH3-SAMHD1-3xHA (21) or plvx-mCherry-SAMHD1-3xHA (Genscript) as a template and mutation-specific primers (IDT). For the experiment using pKH3-3xHA constructs, 4 μg of pKH3-SAMHD1-3xHA plasmids were cotransfected with 4 μg GFP as a transfection control. For plvx-mCherry-3xHA plasmids, 4 μg of plvx-mCherry-SAMHD1-3xHA plasmids were transfected. Both experiments transfected 6-well plates of 293T cells using polyethyleneimine (PEI) (Polysciences, Inc). Transfection media were changed 16 h after transfection. About 48 h after transfection, the cells were washed with PBS and detached using 0.25% trypsin (Corning). Half of each sample was fixed in 4% paraformaldehyde and gated for GFP or mCherry using flow cytometry (MACSQuant VYB with MACSQuantify Software) to determine transfection efficiency (Table S1). The other half of the sample was lysed with cold radioimmunoprecipitation (RIPA) buffer (50 mM Tris–HCl, pH 8, 0.1% SDS, 150 mM NaCl, 0.25% deoxycholic acid, 1 mM EDTA, and 1% NP-40) supplemented with 1:500 (v/v) protease inhibitor cocktail (Sigma). Lysates were clarified using centrifugation (13,000 rpm for 10 min at 4 °C), and the supernatants were stored at −80 °C for immunoblot.

### Immunoblots

Lysates were diluted with Laemmli buffer (Bio-Rad), resolved by SDS-PAGE, and transferred onto a nitrocellulose membrane. These membranes were probed using the indicated primary antibodies and the corresponding secondary antibodies. The membranes were then imaged using SuperSignal West Femto Maximum Sensitivity Substrate (Thermo Fisher Scientific). The following primary antibodies were used during this study: SAMHD1 (abcam; 67820), GAPDH (Cell Signaling; 14C10), HA (Cell Signaling; C23F4), CtIP (Santa Cruz; 271339), cyclin A2 (Abcam; 181591), and FLAG (Abcam 18230). The following secondary antibodies were used during this study: anti-mouse (Cytiva; NA931V) and anti-rabbit (Cytiva; NA934V).

### SAMHD1 protein expression and purification

Plasmids expressing the HD domain (residues 113–626) of SAMHD1 cancer-associated mutations were generated by QuikChange using pet14b-SAMHD1 as a template and mutation-specific primers. The N-terminal His-tagged SAMHD1 proteins were overexpressed in Rosetta DE3 cells (Novagen) by inducing with 0.2 mM IPTG at an optical density of 0.5 to 0.8 for 48 h at 16 °C. Cells were harvested by centrifugation (3500 rpm for 30 min at 4 °C), resuspended in lysis buffer (40 mM Tris–HCl, pH 7.5, 250 mM KCl, 5% glycerol, 0.1% Triton X-100, 5 mM β-mercaptoethanol, and 0.1 mM phenylmethylsulphonyl fluoride), and sonicated. Cleared lysate was obtained by centrifugation (15,000 rpm for 30 min at 4 °C). The cleared lysate was loaded onto a HisTrap FF column (Cytiva) equilibrated with binding buffer (40 mM Tris–HCl, pH 7.5, 10% glycerol, 500 mM NaCl, 5 mM β-mercaptoethanol, and 20 mM imidazole). The column was washed with 75 ml of binding buffer followed by 30 ml of high salt wash buffer (40 mM Tris–HCl, pH 7.5, 10% glycerol, 2000 mM NaCl, 5 mM β-mercaptoethanol, and 20 mM imidazole). Protein was eluted with 50% binding buffer and 50% elution buffer (40 mM Tris–HCl, pH 7.5, 10% glycerol, 300 mM NaCl, 5 mM β-mercaptoethanol, and 500 mM imidazole). Fractions were assessed for the presence of SAMHD1 using SDS-PAGE. Fractions containing SAMHD1 were combined and further purified on HiLoad 16/600 Superdex 200 pg (GE Healthcare) with gel filtration buffer (50 mM Tris–HCl, pH 7.5, 20% glycerol, 150 mM NaCl, and 5 mM β-mercaptoethanol). Fractions were assessed using SDS-PAGE, and those containing SAMHD1 were combined, flash frozen, and stored at −80 °C.

### Thermal shift assay

Thermal shift reaction mixtures (40 μl) were made using 2.5 μM SAMHD1 WT or mutant proteins in SAMHD1 storage buffer (50 mM Tris–HCl, pH 7.5, 20% glycerol, 150 mM NaCl, and 5 mM β-mercaptoethanol), 1 μl SYPRO Orange Protein Gel Stain (Sigma–Aldrich) diluted 1:20 in SAMHD1 storage buffer, and 10 μM dGTP. Reactions were performed in triplicate, and wells containing no SAMHD1 or no dye served as negative controls. Reaction mixtures were added to a 96-well plate (Lightcycler 480 Multiwell Plate 96 white; Roche) and heated from 32 °C to 99 °C at the rate of 0.02 °C per second by a real-time PCR device (LightCycler 480 II; Roche). Florescence was read at 533 to 660, and 30 acquisitions were taken per degree Celsius to monitor protein unfolding signified by increased SYPRO Orange fluorescence. Fluorescent intensity was plotted against temperature using Spyder Software (Anaconda). *T*_m_ was calculated for each well using the transition midpoint.

### Crosslinking-based tetramerization assay

The SAMHD1 crosslinking assay was adapted from a protocol previously described (50). Reaction mixtures (20 μl) contained 10 μM WT or mutant SAMHD1 in crosslinking buffer (50 mM Hepes 7.4, 20% glycerol, 150 mM KCl, and 1 mM β-mercaptoethanol), 250 mM KCl, and 25 mM MgCl_2_. These reactions contained no dGTP or 2 mM dGTP as specified in the text. Reactions were incubated on ice for 30 min followed by incubation at room temperature for 5 min. An equal amount of 2% formaldehyde was added, and the reactions were incubated at 37 °C for 15 min. Reactions were quenched by adding glycine to a final concentration of 250 mM and incubating at room temperature for 15 min. Reactions were diluted with 4× Laemmli buffer without β-mercaptoethanol supplemented and loaded onto an SDS-PAGE gel (Mini-PROTEAN TGX Stain-Free Precast Gel; Bio-Rad). The gel was imaged using ChemiDoc Touch Imaging System (Bio-Rad).

### SAMHD1 degradation assay

Vpx-mediated degradation was performed as previously described (15). Briefly, 1 × 10^6^ 293T cells were cotransfected with 2 μg of pSIV3^+^ or pSIV3^+^ ΔVpx (obtained from Dr Nathaniel Landau, New York University) and 0.1 μg of pKH3-3xHA or pKH3-SAMHD1-3xHA (WT, R366C, or R366H) plasmids by PEI. Transfection media was changed 16 h after transfection. About 48 h after transfection, the cells were washed with PBS and lysed with cold RIPA buffer supplemented with 1:500 (v/v) with a protease inhibitor cocktail (Sigma). Lysates were clarified using centrifugation (13,000 rpm for 10 min at 4 °C), and the supernatants were stored at −80 °C for immunoblot.

### TLC-based dNTPase assay

TLC-based dNTPase assay was adapted from a protocol described previously (31). About 0.02 μM WT or mutant SAMHD1 protein was incubated with 1 μCi/μl [α-32P]dGTP (EasyTide, PerkinElmer), 200 μM unlabeled dGTP (Invitrogen), in TLC reaction buffer (50 mM Tris–HCl, pH 8, 50 mM KCl, 5 mM MgCl_2_, and 0.1% Triton X-100) at 37 °C for 60 min. Reactions were performed in triplicate. This incubation time was chosen because WT displays significant dNTP hydrolysis at this time point, while still being under saturating substrate concentrations during a preliminary time-course experiment. The reactions were heat inactivated by incubating at 70 °C for 5 min. About 0.5 μl of each reaction was spotted onto a PolyGram 300 CEL TLC plate (Macherey–Nagel). The radioactive dGTP substrate and TP product were separated using the mobile phase (800 mM LiCl and 100 mM Tris–HCl at pH 8). Radioactivity was detected using Amersham Typhoon IP (Cytiva), and densitometry analysis was performed using ImageQuant TL 8.2 (Cytiva). Relative activity was determined by dividing TP by the lane total and normalizing to WT. This was repeated for the other dNTP substrates with a modified reaction condition to account for the necessary dGTP activation: 0.04 μM WT or mutant SAMHD1 protein was incubated with 1 μCi/μl [α-32P]dNTP, 200 μM unlabeled dNTP, and 200 μM unlabeled dGTP in TLC reaction buffer at 37 °C for 60 min.

### Crystallization and data collection

Crystals for the HD domain of SAMHD1 (residues 113–626) R366C/H were obtained using catalytically inactive H206R/D207N constructs (29) with the microbatch under oil method. R366C or R366H (4 mg/ml; 50 mM Tris, pH 8.0, 150 mM NaCl, 5 mM MgCl_2_, and 0.5 mM Tris(2-carboxyethyl)phosphine) was mixed with 4 mM dGTP, incubated on ice for 15 min, and added 1:1 with crystallization buffer (100 mM succinic acid–phosphate–glycine buffer, 30% w/v PEG1500; Qiagen). R366C crystallized at pH 8.2 in ∼2 weeks at room temperature. R366H crystallized at pH 9.0 within 1 to 2 days at room temperature. Both constructs were cryoprotected with 25% v/v glycerol and flash frozen in liquid nitrogen. Diffraction data were collected on the NECAT beamline 24-ID-E at the Advanced Photon Source, Argonne National Laboratory. Data statistics are summarized in Table S2.

### Structure determination and refinement

Diffraction images were processed using HKL2000 (66). Structures were solved *via* molecular replacement (Phaser) (67) using PDB 4BZB as a search model. Models were refined through iterative rounds of restrained and TLS refinement (REFMAC5) (68) and model building (Coot) (69). The R366H crystal was twinned with twinning fractions of 0.57 and 0.43 for the (H, K, L) and (H, -K, -H-L) twinning operators, respectively. Residues 278 to 283 were unresolved. Refinement statistics are summarized in Table S2. Coordinate and structure factors were deposited in the PDB under accession codes 7LTT and 7LU5 for R366C and R366H, respectively.

### Generation of U937 cells expressing SAMHD1 mutations

Plasmids expressing SAMHD1 cancer-associated mutations were generated using LPCX-SAMHD1-FLAG as a template and mutation-specific primers. LPCX-FLAG was used as a negative “empty” control. Retroviral vectors were generated as previously reported (32). Transduced U937 cells were selected in 0.4 μg/ml puromycin. Transduction was confirmed by lysing cells in RIPA buffer and performing an immunoblot.

### HIV-1 vector transduction

Recombinant HIV-1 expressing GFP and pseudotyped with the vesicular stomatitis virus G glycoprotein were prepared as described (49). For transductions, 6 × 10^4^ cells seeded in 24-well plates were first treated with 10 ng/ml phorbol-12-myristate-3-acetate for 16 h to induce differentiation into macrophage-like cells. Cells were then transduced with the pseudotyped HIV-1 in the amounts noted in the text. About 48 h after transduction, the percentage of GFP-positive cells was determined by flow cytometry (Becton Dickinson).

### Cellular dNTP measurement

Cellular dNTPs were measured by HIV-1 RT-based dNTP assay as described (54). Briefly, 2 × 10^6^ cells for each cell type were washed with PBS and resuspended in ice-cold 65% methanol. To lyse, cells were vortexed for 2 min and incubated at 95 °C for 3 min. Cells were centrifuged at 14,000 rpm for 3 min, and the supernatant was dried using a speed vac. The dried sample was resuspended in water and diluted to be within the linear range of the assay. 5′ 32P-end-labeled 18-mer DNA primer (5′-GTCCCTCTTCGGGCGCCA-3′; Integrated DNA Technologies) was individually annealed to one of four 19-mer DNA templates (3′-CAGGGAGAAGCCCGCGGTN-5′; Integrated DNA Technologies), where N represents the nucleotide variation at the 5′ end. Reactions (20 μl) contained 200 fmol template/primer, 4 μl of purified RT (HIV-1 HXB2), 25 mM Tris–HCl, pH 8.0, 2 mM dithiothreitol, 100 mM KCl, 5 mM MgCl_2_, and 10 μM oligo (dT), and cellular dNTP extracts. Water or 0.5 mM dNTP mix was used as a negative and positive control, respectively. Reactions were incubated at 37 °C for 5 min and stopped by adding 10 μl 40 mM EDTA and 99% (v/v) formamide followed by incubation at 95 °C for 2 min. Reactions were resolved on a 14% urea-PAGE gel (AmericanBio, Inc) and analyzed using PharosFX molecular imager. Image Lab software, version 5.1.2, (Bio-Rad) was used to quantify single-nucleotide extensions products.

### Immunoprecipitation

293T cells in 10-cm dishes were transfected with pKH3-3xHA or pKH3-SAMHD1-3xHA (WT, R366C, or R366H) using PEI. Transfection media was changed 16 h after transfection. About 48 h after transfection, the cells were lysed in immunoprecipitation lysis buffer (25 mM Tris–HCl, 50 nM NaCl, 5% glycerol, and 0.375% Chaps) supplemented with 1:500 (v/v) protease inhibitor cocktail (Sigma) and centrifuged to obtain cleared lysate (14,000 rpm for 5 min at 4 °C). Monoclonal Anti-HA Agarose (Sigma) was washed three times in RIPA buffer, added to the cleared lysate, and incubated overnight at 4 °C with gentle rocking. The next day, the sample was centrifuged (3000*g* for 2 min at 4 °C) to pellet the resin and bound protein. The supernatant was discarded, and the resin was loaded onto HA spin column. The column was washed three times with RIPA buffer supplemented with protease inhibitor cocktail. The remaining bound protein was eluted with 8 M urea (Sigma) and stored at −80 °C for immunoblot.

### Double-strand break reporter assay

Measurement of dsDNA break repair by homologous recombination was conducted as reported previously (21). Briefly, U20S cells stably expressing a DR-GFP reporter gene were transfected with 30 nM siRNA using Lipofectamine RNAiMax. The following day, transfection media was changed, and cells were transfected with I-SceI and RFP or SAMHD1-RFP (WT, R366C, or R366H) plasmids. Cells were harvested 72 h after transfection of plasmids. RFP-expressing cells were gated and analyzed for homologous recombination based on GFP expression using fluorescence-activated cell sorting. This experiment was performed in quadruplicate (n = 4).

### LTR and ISRE luciferase assays

293T cells were seeded in 12-well plates. For LTR suppression, cells were transfected with 100 ng pBlue3′LTR-luc-C (National Institutes of Health [NIH] AIDS: #4789), 100 ng Renilla luciferase, and 300 ng pKH3-3xHA or pKH3-SAMHD1-3xHA (WT, R366C, or R366H) using PEI (25). For ISRE suppression, cells were transfected with 100 ng ISRE-Luc (obtained from Li Wu, Ohio State University), 100 ng Renilla luciferase, 200 ng IRF7-FLAG (obtained from Li Wu, Ohio State University), and 300 ng pKH3-3xHA or pKH3-SAMHD1-3xHA (WT, HD/RN, R366C, or R366H) using PEI (23). About 16 h later, transfection media were changed to fresh media. About 24 h after transfection, cells were lysed and assessed for luciferase activity using the Dual-Luciferase Reporter Assay System (Promega) following the manufacturer's instructions. The remaining lysate was stored at −20 °C for immunoblot. LTR and ISRE activity was calculated by dividing firefly luciferase activity by Renilla luciferase activity and normalizing to the empty vector condition. The immunoblot was quantified using Image Lab Software (Bio-Rad) (Fig. S3). SAMHD1 signal/GAPDH signal was normalized to the WT condition. The previous luciferase calculation was divided by the normalized Western blot signal in order to account for variations in expression.

### Oligonucleotide binding by fluorescence polarization

WT and mutant variants of HD-domain SAMHD (114–626) constructs were expressed using a bacterial expression system and purified, as previously described (70). The binding of SAMHD1 to various oligonucleotides was monitored by fluorescence polarization. The reporter oligonucleotides containing a 6-carboxyfluoroscein label at the 5′ end were purchased from IDT. ssDNA57 and ssRNA40 oligonucleotides had the same sequences as in the original study (27). The binding assay contained 50 nM of fluorescently labeled oligonucleotide and was prepared in the following buffer: 50 mM Tris–HCl, pH 8.0, 50 mM KCl, and 1 mM EDTA. Fluorescence polarization measurements were performed in 384-well plates (Corning; 3575) on a Synergy 2 microplate reader (Biotek) using 485/20 nm excitation and 530/20 nm emission bandpass filters. All experiments were performed in duplicate with 20 μl of solution volume in each well.

### VLP transduction of CD4^+^ T cells

VLPs Vpx^+^ and Vpx^−^ were prepared as previously described (16). Briefly, 293T cells were transfected with 40 μg of pSIV3^+^ or pSIV3^+^ ΔVpx (obtained from Dr Nathaniel Landau, New York University) and 20 μg of pVSV-G using PEI. Supernatant was collected 2 and 3 days post-transfection and centrifuged to clear cellular debris (1200 rpm for 7 min). Cleared supernatant was overlaid above a 25% sucrose solution (25% sucrose, 25 mM Tris–HCl, pH 7.5, 150 mM NaCl, and 5 mM EDTA) and concentrated by ultracentrifugation (23,000 rpm, for 2 h at 4 °C). Pellets were resuspended in Hanks' balanced salt solution (GIBCO), aliquoted, flash frozen, and stored at −80 °C. Activated CD4^+^ T cells were transduced with either VLP Vpx^+^ or VLP Vpx^−^. Cells were harvested for Western blot and dNTP measurements 24 h after transduction.

### Statistical analysis

All statistical analyses were conducted using two-tailed and unpaired Welch's *t* tests. *p* values obtained from each analysis are included in the figure. The cutoff for statistical significance in this study is a *p* value < 0.05. All experiments were performed in triplicate (n = 3) unless otherwise noted. Values are reported as the mean of these replicates, and error bars in each figure represent standard deviation.

## Data availability

The atomic coordinates and structure factors have been deposited in the PDB. SAMHD1 (113–626) H206R/D207N/R366C—PDB ID: 7LTT. SAMHD1 (113–626) H206R/D207N/R366H—PDB ID: 7LU5.

## Supporting information

This article contains supporting information.

## Conflict of interest

The authors declare that they have no conflicts of interest with the contents of this article.
