# Supplementary material for: Structural and functional characterization explains loss of dNTPase activity of the cancer-specific R366C/H mutant SAMHD1 proteins
Source: J Biol Chem. 2021 Sep 4;297(4):101170. doi: 10.1016/j.jbc.2021.101170 (PMC8497992; doi:10.1016/j.jbc.2021.101170)
Supplement: Tables S1 and S2; Figures S1–S3 [file mmc1.pdf]

a

Condition	%GFP Positive Cells
WT	28.72%
Empty	25.24%
R145Q	29.84%
Y155C	38.16%
P158S	35.66%
I201N	42.69%
R366C	37.47%
R366H	21.81%

b

Condition	%mCherry Positive Cells
WT	69.2%
Empty	86.4%
L244F	68.9%
R451C	93.6%
F578V	72.2%

Supporting Table 1: Transfection efficiency of SAMHD1 cancer mutants. 293T cells were transfected with SAMHD1 plasmids and a GFP plasmid (left) or an mCherry labelled SAMHD1 plasmid (right). Half of the sample was fixed and analyzed for GFP or mCherry using flow cytometry to determine transfection efficiency.

	R366C	R366H
Data collection		
Wavelength (Å)	0.97918	0.97918
Space group	$P2_1$	$P2_1$
Cell dimensions<br>a, b, c (Å)<br>α, β, γ (˚)	 80.9, 140.1, 97.2<br>90.0, 114.2, 90.0	 83.7, 573.5, 100.5<br>90.0, 114.7, 90.0
No. molecules/asymmetric unit	4	16
Resolution (Å)	50.0-1.9 (1.93-1.90)	50.0-3.60 (3.66-3.60)
R_{merge}	0.071 (>1)	0.143 (>1)
Mean I / σI	17.3 (1.0)	13.8 (1.6)
$CC_{1/2}$	0.999 (0.297)	0.996 (0.690)
Completeness (%)	99.1 (98.8)	85.6 (83.1)
Redundancy	3.3 (3.3)	5.1 (4.8)
Unique reflections	153,730 (7,622)	84,897 (4,131)
Refinement		
No. nonhydrogen atoms	16,683	64,032
R_{work}/R_{free}	0.173/0.207	0.227/0.259
Mean B-factor ($Å^2$)	31	144
R.m.s.d.<br>Bond lengths (Å)<br>Bond angles (˚)	 0.012<br>1.7	 0.008<br>1.4
Ramachandran<br>Favored (%)<br>Allowed (%)<br>Outliers (%)	 98.64<br>1.26<br>0.10	 97.84<br>2.11<br>0.05

Supporting Table 2: Crystal Structure data collection and refinement statistics.

a

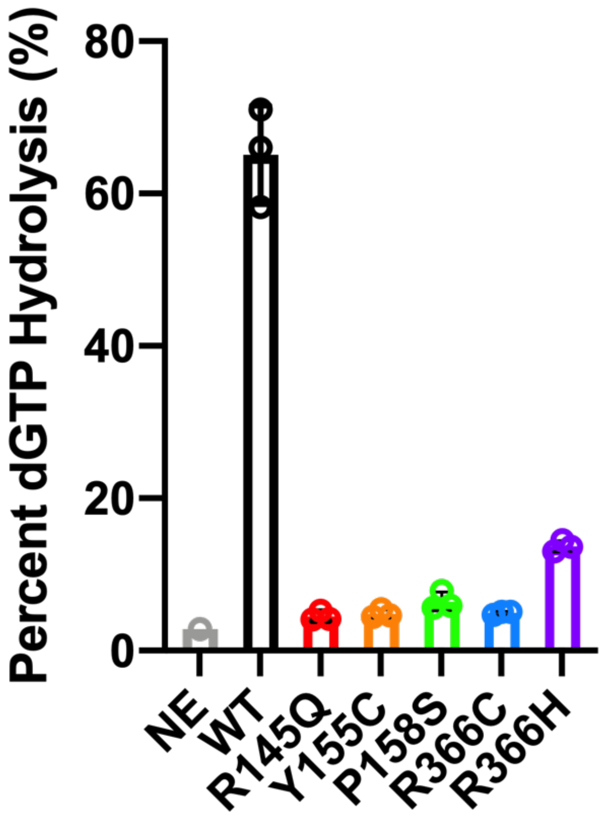

Supporting Figure 1: Percent dGTP hydrolysis of SAMHD1 mutants. Wild type and mutant SAMHD1 were incubated with α^{32}P-dGTP in the presence of excess unlabeled dGTP. Reaction substrate and product were separated using TLC (Figure 3A). Densitometry analysis of the TLC plate was performed and percent dGTP hydrolysis was calculated by dividing the triphosphate product by the lane total.

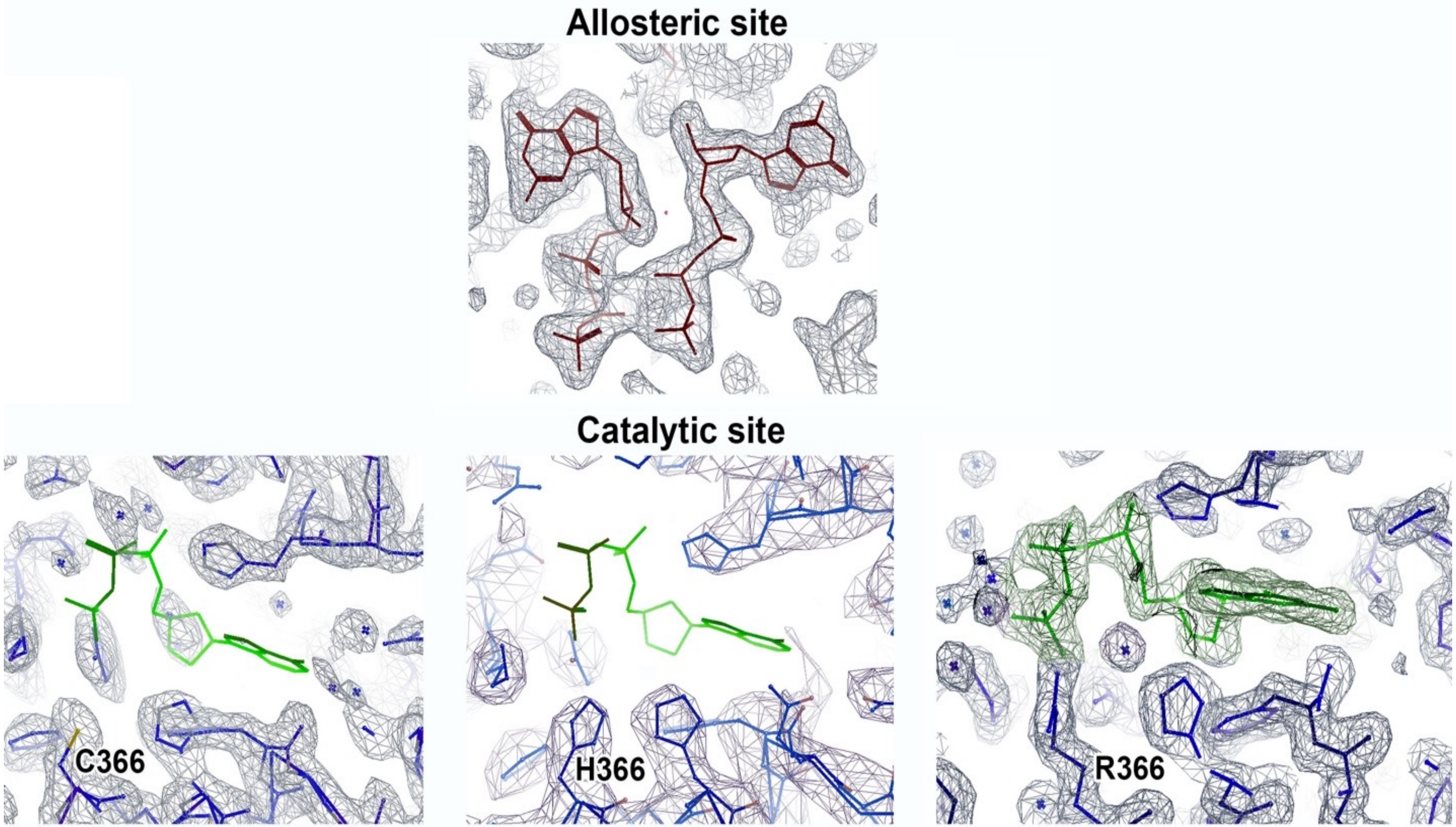

Supporting Figure 2: Electron density in the allosteric and catalytic sites of R366C/H. 2Fo-Fc electron density (1s level, gray mesh) of the regions around the allosteric nucleotides of the R366C structure (top) and the catalytic site of the R366C (bottom left), the R366H (bottom middle, sharpened by a B-factor of -80 Å^2), and the SAMHD1$_{HD/RN}$ (bottom right, PDB 4BZB) structures. There is a lack of nucleotide density in the catalytic site of the R366C/H mutant structures, where the nucleotide substrate (green) was modeled in based on its position in the SAMHD1$_{HD/RN}$ structure.

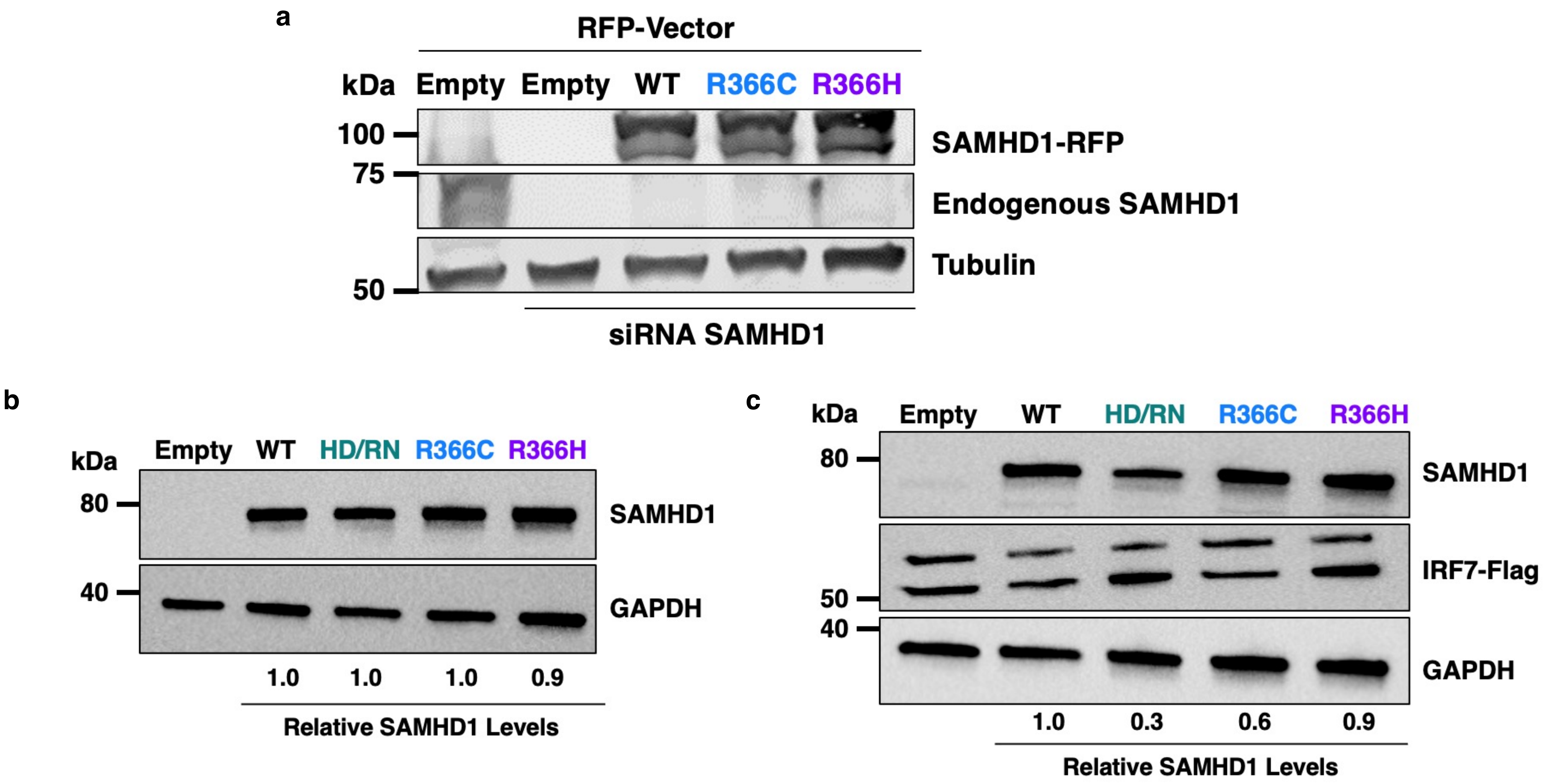

Supporting Figure 3: Immunoblot for SAMHD1 expression levels. a. dsDNA repair by Homologous Recombination **b.** LTR-Luciferase assay **c.** ISRE-Luciferase assay. Expression levels of SAMHD1 were used to normalize activity data in Figure 6.
